# Reconstructing ribosomal genes from large scale total RNA meta-transcriptomic data

**DOI:** 10.1093/bioinformatics/btaa177

**Published:** 2020-03-13

**Authors:** Yaxin Xue, Anders Lanzén, Inge Jonassen

**Affiliations:** b1 Computational Biology Unit, Department of Informatics, University of Bergen, Bergen, Norway; b2 AZTI-Tecnalia, Herrera Kaia, 20110 Pasaia, Spain; b3 Ikerbasque, Basque Foundation for Science, 48011 Bilbao, Spain

## Abstract

**Motivation:**

Technological advances in meta-transcriptomics have enabled a deeper understanding of the structure and function of microbial communities. ‘Total RNA’ meta-transcriptomics, sequencing of total reverse transcribed RNA, provides a unique opportunity to investigate both the structure and function of active microbial communities from all three domains of life simultaneously. A major step of this approach is the reconstruction of full-length taxonomic marker genes such as the small subunit ribosomal RNA. However, current tools for this purpose are mainly targeted towards analysis of amplicon and metagenomic data and thus lack the ability to handle the massive and complex datasets typically resulting from total RNA experiments.

**Results:**

In this work, we introduce MetaRib, a new tool for reconstructing ribosomal gene sequences from total RNA meta-transcriptomic data. MetaRib is based on the popular rRNA assembly program EMIRGE, together with several improvements. We address the challenge posed by large complex datasets by integrating sub-assembly, dereplication and mapping in an iterative approach, with additional post-processing steps. We applied the method to both simulated and real-world datasets. Our results show that MetaRib can deal with larger datasets and recover more rRNA genes, which achieve around 60 times speedup and higher F1 score compared to EMIRGE in simulated datasets. In the real-world dataset, it shows similar trends but recovers more contigs compared with a previous analysis based on random sub-sampling, while enabling the comparison of individual contig abundances across samples for the first time.

**Availability and implementation:**

The source code of MetaRib is freely available at https://github.com/yxxue/MetaRib.

**Contact:**

yaxin.xue@uib.no or Inge.Jonassen@uib.no

**Supplementary information:**

Supplementary data are available at *Bioinformatics* online.

## 1 Introduction

Advances in next-generation sequencing have boosted the study of microbial communities in many ecosystems. Meta-transcriptomics, the direct sequencing and analysis of all RNA in a microbial community, has been widely used in investing microbial universe from various environments (Carvalhais *et al.*, 2012; Jorth *et al.*, 2014; Shi *et al.*, 2009). It provides an informative perspective about the current state of functional output, as it can elucidate which members and functions of a community are active in certain circumstances, rather than only the genomic contents (Franzosa *et al.*, 2015). Meta-transcriptomics is considered to be more efficient in observing rapid regulatory responses than meta-proteomics (Carvalhais *et al.*, 2012). Moreover, it could capture the information missing in DNA-based metagenomics, such as RNA viruses (Culley, 2006; Zhang *et al.*, 2006). The whole microbial RNA pool is dominated by rRNA and tRNA (95–99%), while only small fractions are mRNA (1–5%) (Carvalhais *et al.*, 2012). To date, most meta-transcriptomic studies have focused on function (mRNA) rather than structure, depleting rRNA both experimentally and *in silico*.

‘Total RNA meta-transcriptomics’ involves the isolation and sequencing of reverse transcribed total RNA pools—including mRNA (gene expression), rRNA (abundance), RNA viruses, tRNA and other non-coding RNA—from samples without any PCR or cloning step. In contrast to normal meta-transcriptomics, this approach enables us to obtain both structural and functional information simultaneously in a microbial community (Urich *et al.*, 2008). It answers two fundamental questions in microbial research—‘who is there?’ and ‘what are they doing?’—with a few advantages. In terms of structural investigation, total RNA meta-transcriptomics assesses taxonomic diversity in all three domains of life, meanwhile avoiding amplification bias, compared to PCR-based amplicon surveys. Ribosomal RNA is also essential for protein synthesis in all organisms. Thus, its relative abundance across taxa generally reflects the overall structural activity in a community. For functional profiling, it provides novel insights into current gene activity status with corresponding structural profiling simultaneously in one experiment.

Several tools are available for meta-transcriptomics, e.g. IMP (Narayanasamy *et al.*, 2016), SAMSA (Westreich *et al.*, 2016), MetaTrans (Martinez *et al.*, 2016), but they are geared mainly for studying the functional profiling. Though typically disregarded in meta-transcriptomics, rRNA and its corresponding gene is widely used as a genetic marker to study bacterial phylogeny and taxonomy, as it is present in all domains and has both highly conserved regions and regions that vary between species. Currently, most structural rRNA profiling relies on amplicon sequencing (meta-barcoding) using ‘universal’ primers to target and amplify hypervariable regions of rRNA or other taxonomic markers as broadly as possible (Rosselli *et al.*, 2016). Although amplicon sequencing represents a fundamentally important method for studying microbial and other biological communities, it is susceptible to biases depending on the specificity/universality of the primers used and other PCR conditions. Thus, it may lead to an incomplete or biased profile of the true biodiversity present in a given sample (Lanzén *et al.*, 2011; Shakya *et al.*, 2013). By using total RNA meta-transcriptomics for structural profiling, such biases can be avoided. Furthermore, it allows for the reconstruction of full-length rRNA sequences, enabling a higher resolution for taxonomy profiling. This is typically not feasible in meta-barcoding; using short-read sequencing technologies results in amplicons with insufficient phylogenetic signal, while long-read sequencing allow for longer amplicons but is currently restricted by higher error rates. Existing *de novo* assembly tools for shotgun sequence reads are designed primarily for genomic or metagenomic data and do not perform well on rRNA genes (Yuan *et al.*, 2015). Instead, there are several tools developed specifically for rRNA recovery and assembly, such as EMIRGE (Miller *et al.*, 2013), REAGO (Yuan *et al.*, 2015), RAMBL (Zeng *et al.*, 2017) and MATAM (Pericard *et al.*, 2018). However, these tools were designed for analysis of smaller datasets and cannot be used directly to analyze total RNA meta-transcriptomics studies.

Here, we present MetaRib, a novel tool for constructing full-length ribosomal gene sequences optimized for total RNA meta-transcriptomic data. Firstly, its dereplication process enables us to identify both existing species and novel species, while minimizing false positives. Furthermore, it significantly reduces the running time and memory usage by an iterative sampling approach, making it possible to assemble rRNA sequences from very large datasets: combining several samples also allows for reconstructing rRNA from less abundant species. Thus, MetaRib allows us to study the distributions of assembled rRNA sequences across multiple samples, independent of taxonomical classification. This is done by mapping reads to the resulting assembled small subunit ribosomal RNA (SSU rRNA) sequences, which we consider as operational taxonomic units (OTUs).

Our approach exploits the uneven taxon-abundance distribution common for microbial communities, with few dominating taxa and a long tail of rarer ones, often referred to as the ‘rare biosphere’ (Sogin *et al.*, 2006).

In practice, this leads to high redundancy in total RNA meta-transcriptomic data, with many sequences originating from the most abundant species. Our assumption is that rRNA of highly abundant species can be reconstructed from a relatively small subsample of the sequences. Subsequently, all rRNA sequences in the whole dataset related with the same species could be removed from further analysis, enabling reconstruction of less abundant species iteratively. Merging reads from several samples or datasets can also help to reconstruct rarer species, below the assembly threshold in smaller datasets. We evaluated our tool using three simulated total RNA datasets (limited to prokaryotic rRNA with special design to access different scenarios) and benchmarked its performance. Moreover, a real-world dataset from a large-scale soil total RNA experiment consisting of three billion SSU rRNA reads was analyzed, showing that MetaRib could recover more information than what was possible in the previous study of the same data.

## 2 Materials and methods

### 2.1 Metarib workflow

**Fig. 1.**
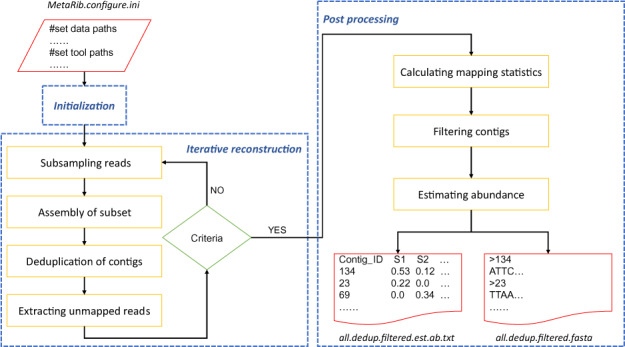
Schematic overview of MetaRib workflow. Dash rectangles depict main modules, solid line rectangles represent major steps in each module and red elements denote input and output files

The MetaRib algorithm consists of three major modules: (i) initialization, (ii) iterative reconstruction and (iii) post-processing, summarized in Figure 1.

#### 2.1.1 Initialization

A configuration file is needed to initiate the workflow, which first controls the availability of data and standalone software tools (dependencies). A case-specific workflow script is then generated and executed. A full description of the input configuration file and data structure is found in the GitHub repository (https://github.com/yxxue/MetaRib).

#### 2.1.2 Iterative reconstruction

MetaRib uses an iterative process to reconstruct rRNA contigs. The workflow is initiated on a randomly picked subset of the total reads, which are assembled, and used to filter remaining reads by removing those that can be mapped perfectly to the resulting contigs. This process (random selection, assembly and filtering) is then repeated until a pre-defined termination criterion is reached. This module is composed of five steps:

*Step 1: Subsampling reads*

The first step is initial subsampling of sequencing data from the remaining unmapped reads. In each iteration, a subset of *n* reads (provided in the configuration file, by default *n *=* *100 000) is randomly picked from the total unmapped reads **U**, of size *N* (initially containing all reads). MetaRib will change the seed number automatically at each iteration to avoid repetitive sampling of reads.

*Step 2: Assembly of subset*

The randomly picked subset of size *n* is used as input to EMIRGE (Miller *et al.*, 2013) for reference-assisted assembly into rRNA contigs. The EMIRGE assembly parameters, including the reference sequence database used, can be specified in the configuration file. Considering that the community structure is relative uneven, for most natural communities, contigs corresponding to highly abundant species are more likely to be assembled in the first several iterations even when *n ≪ N*.

*Step 3: Dereplication of contigs*

When the assembly is completed, contigs resulting from Step 2 of the current iteration are compared with the existing assembled rRNA contig set **C** (initially empty). New contig sequences are first concatenated to existing ones, then sorted by sequence length and renamed with unique IDs. Overlap-based clustering is then performed to eliminate duplicated and keep longest contig sequences for each cluster using a stringent threshold, considering the high similarity of rRNA contigs.

*Step 4: Mapping of remaining unmapped reads*

All unmapped reads, **U** (i.e. all reads in the first iteration) are aligned against the dereplicated contig set **C** using stringent parameters, considering the presence of highly conserved regions in the rRNA gene. Reads that align to the contigs are removed from **U**, leaving only unmapped reads for subsequent iterations. Since sequences from highly abundant taxa are more likely to be assembled in the first iterations, a large proportion of raw reads are likely to be removed, which facilitates assembly of remaining reads. This is the key approach of MetaRib to reduce the complexity, memory and time requirement when assembling large datasets from typical, uneven biological communities.

*Step 5: Terminating criteria*

The iterative process will be terminated under three circumstances: (i) it reaches a maximum of 11 iterations; (ii) the remaining unmapped reads is less than *n*; or (iii) the last iteration produced a sufficiently small number of novel contigs (<1% of the current contig set). The last situation may indicate that assembly from a subsample of size *n* is difficult due to poor coverage of all taxa present. To counteract this, a final extra iteration is carried out using a subsample of size *2n*.

#### 2.1.3 Post-processing

Once any of the criteria for halting the iteration have been met, a final non-redundant contig set is generated. MetaRib will then start post-processing to filter out low-quality contigs and estimate their relative abundance across individual samples.

*Step 1: Calculating mapping statistics*

Raw reads from each sample are aligned to the contig set **C** to generate several mapping statistics by BBMAP, including the mapping rate (%), coverage and covered percentage of each particular contig in **C**.

*Step 2: Filtering contigs*

Low-quality contigs are filtered by parsing mapping statistics report from Step 1. We consider a particular contig in **C** is a false positive record if either its average coverage or percent of bases covered are below a pre-defined threshold (by default 2 and 80%, respectively).

*Step 3: Estimating abundance*

The mapping rate is used to represent relative abundance of contigs in each sample. As rRNA genes contain both conserved and variable regions, we choose to include both ‘unambiguous’ mapping (where a merged read is aligned to only one contig) and ‘ambiguous’ mapping (where a read can be aligned to more than one contig).

Finally, MetaRib will generate two files: one containing the high-quality contig sequences (in FASTA format) and one matrix (‘OTU table’) that summarizes the abundance information across samples, which each row representing a contig and each column representing a sample. These numbers are assumed to approximate the abundances of taxa corresponding to the reconstructed contigs. An exception is species with considerable intra-specific rRNA sequence variation, for which total abundance instead can be obtained by identifying and adding the relative abundances for their contigs.

### 2.2 Implementation

MetaRib is developed with Python2.7 and is distributed under the GNU GPL v3.0 license. MetaRib is freely available on https://github.com/yxxue/MetaRib. Dependencies include the Python libraries Pandas (used for data analysis).

MetaRib also requires EMIRGE for rRNA assembly. EMIRGE was chosen by default as it is one the most widely used for reconstructing full-length rRNA genes and has shown better performance than other methods.

The BBtools suit (https://jgi.doe.gov/data-and-tools/bbtools/) is also required for MetaRib and utilized for several tasks including read mapping and dereplication. BBtools/reformat.sh is used for format conversion and subsampling. BBtools/dedupe.sh is an overlap-based dereplication tool allowing a specified number of substitutions or edit distance, applied in MetaRib’s dereplication step. Default dedupe.sh parameters are maximum five indels and minimum 99% similarity *(fo = t ow = t c = t mcs = 1 e = 5 mid = 99*). BBtools/bbmap.sh is used to map (align) reads to contigs. BBMap has a few advantages for our implementation, including output of unmapped reads immediately (bypassing SAM/BAM format output), which accelerates the iteration process. Furthermore, it performs global rather than local alignment that can avoid excluding excessive reads due to highly conserved regions of rRNA genes. In addition, it returns detailed mapping statistics, used in post-processing. Default parameters for BBMAP is *minid = 0.96 maxindel = 1 minhits = 2 idfilter = 0.98* and users can modify those parameters in the configuration file.

The BBtools suits and EMIRGE need to be installed before MetaRib, and their parameters defined in the configuration file.

### 2.3 Evaluation with simulated datasets

#### 2.3.1 Generation of simulated datasets

**Fig. 2.**
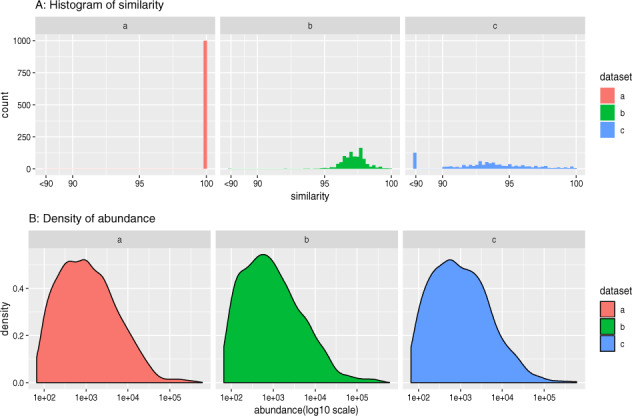
Overview of simulated datasets. (**A**) The global pairwise similarity distribution of picked contigs aligning to the reference is shown. (**B**) Contig abundance distribution for simulated reads in the three test datasets is shown

To simulate the complexity of real microbiome communities, three *in silico* simulated datasets were built. As a full-length rRNA reference dataset, we used the SILVA SSU rRNA reference database (Quast *et al.*, 2012) (release 123). To simulate sequence reads for dataset **a**, one thousand full-length sequences were randomly picked from a version of the reference database clustered at 94% identity using maximum linkage. These reference sequences were used to simulate 5 million Illumina pair-end sequencing reads following a log-normal abundance distribution, using ART (Huang *et al.*, 2012). For Dataset **b**, we randomly selected 1000 sequences from the full non-redundant version of Silva v123 (i.e. not clustered using 94%). Only full-length sequences with a similarity between 95% and 99% to the clustered reference database were retained and used to generate 5 million sequence read pairs with ART following the same distribution. Finally, Dataset **c** was similar with **a**, but all full-length sequences used to generate them were removed from the reference database used by EMIRGE during assembly. An overview of simulated datasets is shown in Figure 2. The intra-dataset sequence similarity was evaluated by performing global all-against-all alignment for each dataset (exclude self-alignment) with minimum pairwise identity 90 (Supplementary Fig. S1). All simulated datasets and corresponding EMIRGE references are deposited at NIRD research data archive (https://archive.sigma2.no/pages/public/datasetDetail.jsf?id=10.11582/2019.00040).

#### 2.3.2 Evaluating performance

Simulated datasets were used to compare the performance of MetaRib with EMIRGE (run non-iteratively). All programs were tested on the same computer cluster using 40 cores (in-house compute server, 80 cores, 1 TB RAM). For the running time comparison benchmark, we used the GNU ‘time’ command to capture both real (elapsed), system and user time.

For the simulated datasets, we could assess the correctness of the reconstructed contigs, i.e. how similar each reconstructed contig was to the ‘source contig’, recording for each reconstructed contig the similarity of the closest source sequence, and vice versa; for each source sequence, the similarity of the closest reconstructed contig. For this analysis, we used Vsearch (Rognes *et al.*, 2016) for performing pair-wise global alignment with 90% minimum identity.

For a range of similarity thresholds, we then counted statistical measures of the performance of two methods*. True positives (TP)* correspond to the number of ‘correctly’ reconstructed contigs (having a reconstructed contig with similarity above the threshold) and *false positives (FP)*—the number of reconstructed incorrect contigs (below the similarity threshold). *False negatives (FN)* correspond to the number of un-reconstructed sequences in the source contig. Finally, we calculate *Precision*, *Sensitivity* and *F1-score* based on the number of *TP*, *FP* and *FN*.

To evaluate the accuracy of abundance estimation, we then performed Pearson’s correlation test between the real abundance of source contigs with the abundance output of the closest reconstructed contigs.

### 2.4 Real-world dataset

In order to evaluate the performance of MetaRib on real-world total RNA sequence data, we utilized the data 3 billion sequence reads generated as part of the AshBack project (Bang-Andreasen *et al.*, 2020). Bang-Andreasen *et al.* (2020) conducted a large-scale total RNA meta-transcriptomic study to access the impact of wood ash on agricultural and forest soil microbial communities and functional expression simultaneously applying four doses of wood ash concentration: 0, 3, 12 and 90 t ha^−1^ (Conc: 0, 3, 12, 90). Each dose was applied to two soil types: agricultural and forest soil and total community RNA extracted and sequenced after 0, 10, 30 and 100 days of incubation (D0, D3, D30, D100). The large-scale and complexity made it an ideal case to apply MetaRib.

A total of 325 Gb rRNA sequences were collected from the wood ash dataset (PRJNA512608). Due to the lack of bioinformatic tools and computational constraints, previous rRNA analysis was performed on a small subset (1.5 million randomly selected sequences) of each sample, using EMIRGE (Bang-Andreasen *et al.*, 2020). We reanalyzed the complete dataset using MetaRib with default parameters, and, in a repeated analysis with *n = 1 000 000* considering the larger size of the dataset. Downstream analysis was performed with Phyloseq (McMurdie and Holmes, 2013) and DADA2 (Callahan *et al.*, 2016), figures were generated using ggplot2 (Ginestet, 2011) and ComplexHeatmap (Gu *et al.*, 2016). Since all samples were analyzed together in MetaRib, we could also detect the presence (here defined as a relative abundance ≥1e−5) of contigs across samples.

## 3 Results

### 3.1 Run time comparison

Table 1 shows statistics of time usage when analyzing the three simulated datasets using EMIRGE non-iteratively and with MetaRib (otherwise using the same parameters). MetaRib could assemble simulated datasets (5 million sequences each) in a few minutes while EMIRGE needs days to run, representing around 60X speedup compared to using EMIRGE out of the box with the same parameters.

**Table 1.** Comparison of programs running time

	User (s)		Elapsed (HH:MM:SS)		Iterations
	EMIRGE	MetaRib	EMIRGE	MetaRib	MetaRib
**a**	2224330	19278	37:02:26	0:28:27	5
**b**	3913996	59468	57:36:44	1:07:30	5
**c**	2499052	45901	37:45:27	0:47:58	7

*Note*: User is the amount of CPU time spent; elapsed is the time from start to finish the program. Iteration is the iteration number in MetaRib for each dataset.

### 3.2 Correctness

**Fig. 3.**
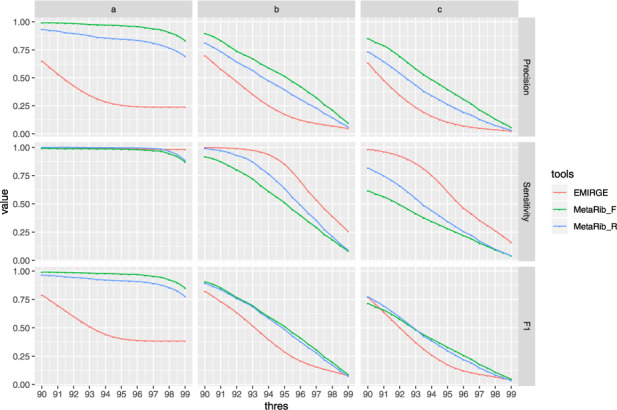
Overview evaluation of correctness. The *X*-axis represents different similarity thresholds used to determine if a reconstructed contig is correct. The *Y*-axis represents the value of measurements (precision, sensitivity and F1-score). MetaRib_F represents contigs filtered with low-quality records, while MetaRib_R is the original output

The relative performance of two tools is shown in terms of *Precision*, *Sensitivity* and *F1-score* for all three simulated datasets representing different scenario. MetaRib shows the best overall performance in all datasets with *F1-score* evaluation (Fig. 3 and Supplementary Table S1). EMIRGE recovers almost all source sequences if they are represented in the reference (Dataset **a**). For **b** and **c**, where source sequences are less similar to the reference database, EMIRGE has a higher sensitivity compared to MetaRib. However, as shown in Figure 3, EMIRGE is also producing a large number ‘false’ contigs, which leads to a quite low precision and F1-score even in an ideal case (Dataset **a**). Conversely, MetaRib is producing far fewer such ‘false’ sequences. We also test the performance of the ‘contig filtering’ step done as part of the post-processing.

Our results demonstrate that filtering low-quality contigs using mapping statistics (MetaRib_F) improves the performance compared with the unfiltered result (MetaRib_R; see 2.1.3 step 3). More detailed results—like statistical metrics of contigs length in each iteration (Supplementary Table S2) and comparison of contigs length distribution between tools (Supplementary Fig. S2)–can be found in the Supplementary Data.

### 3.3 Abundance estimation

**Fig. 4.**
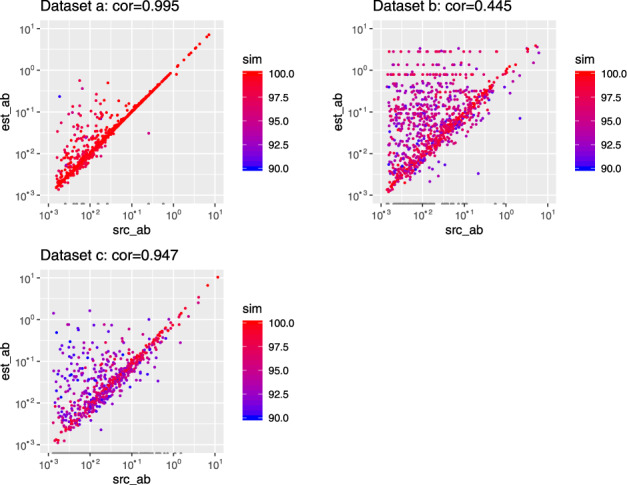
Evaluation of abundance estimation. Values for real abundance (src_ab) and the closest estimated abundance (est_ab) displayed on log–log coordinates and colored with the similarity score (sim). Pearson correlation is calculated between src_ab and est_ab

Figure 4 shows the scatter plot of comparation of relative abundance between source contigs(src_ab) with the closest reconstructed contigs (est_ab). MetaRib could estimate the relative abundance accurately when the nearly full-length contigs are reconstructed (sim ≥ 97.5), even for very low-abundant records (src_ab ≤ 1e−2). As we expected, it has the best performance in an ideal scenario (Dataset **a**); however, it comes up with over-estimation problem at low-abundant records caused by ‘ambiguous’ mapping of conserved region in rRNA sequences which are distinct from the reference (Dataset **b**).

### 3.4 Real-world dataset

MetaRib could complete the analysis of 320 Gb (3 billion reads) in approximately 1–2 days using default parameters with 80 cores. However, the CPU and run time is nearly doubled when using a larger reads sampling number (*n* = 1 000 000; Table 2).

**Table 2.** Comparison of MetaRib running time with different sampling reads number

Sampling_num (*n*)	User (s)	Elapsed (HH:MM:SS)
100 000 (100 K)	7 927 023	38:00:48
1 000 000 (1 M)	12 330 130	62:16:55

*Note*: The program is performed with 80 cores.

**Fig. 5.**
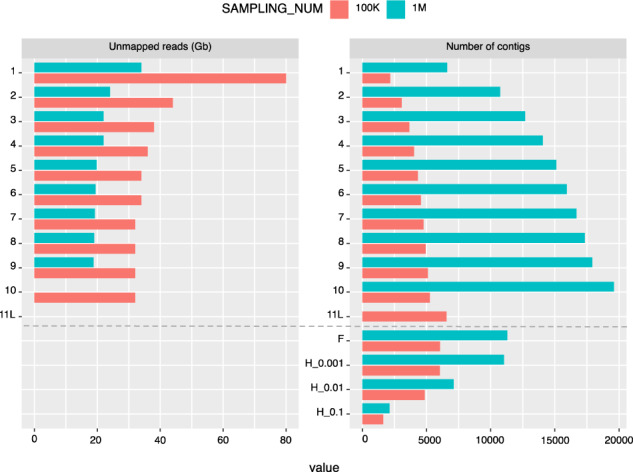
MetaRib performance for ASHBACK dataset per iteration with two different read subsampling numbers *n*. 1–11L: iterations. F: filtered contigs. H: contigs which relative abundance is higher than certain thresholds (0.001%: H_0001, 0.01%: H_0.01, 0.1%: H_0.1) in at least one sample

The read subsampling number *n* also effects the performance of MetaRib, both for the iteration process and final result resulting in 11 iterations for the default value (*n = 100k*) and 9 iterations for *n = 1M* (see Fig. 5). As we expected, the size of **U** decreases significantly in the first few iterations and thus becomes stabilized; while smaller values of *n* need more iterations to converge and result in more remaining unmapped reads after the last iteration. However, larger *n* values also result in more potential false positives. For example, the size of **U** ceases to decrease after five iterations, whereas the number of C maintains a continuous increase. Particularly, nearly half of the **C** fail to pass the filter step (F) using *n = 1M*. We further check the number of contigs which relative abundance is higher than certain thresholds (0.001%: H_0001, 0.01%: H_0.01, 0.1%: H_0.1) in at least one sample according to their estimated abundance. We find that the number of ‘dominant’ (high abundance) contigs using the default value (*n = 100k*) gives closer results to *n = 1M* with a higher threshold, which indicates that the smaller, default value of *n* was sufficient to reconstruct the majority rRNA contigs in a complex community. Results obtained using *n = 1M* were thus excluded from further analysis.

**Fig. 6.**
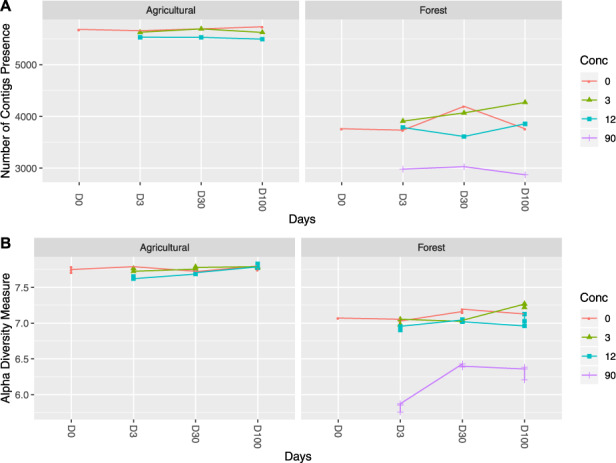
Number of contigs and Shannon diversity across the two soils at increasing wood ash amendment and incubation times (*n* = 100 K). The presence of contig is determined by the average abundance within each measure and soil (≥1e−5). Shannon diversity is estimated based on relative abundance table

We observe more rRNA contigs in both sites and similar trends of richness and Shannon diversity across treatments in forest soil as those revealed by previous analysis (Bang-Andreasen *et al.*, 2020), except considerably less fluctuation of diversity across treatments and time in agricultural soil (Fig. 6).

**Fig. 7.**
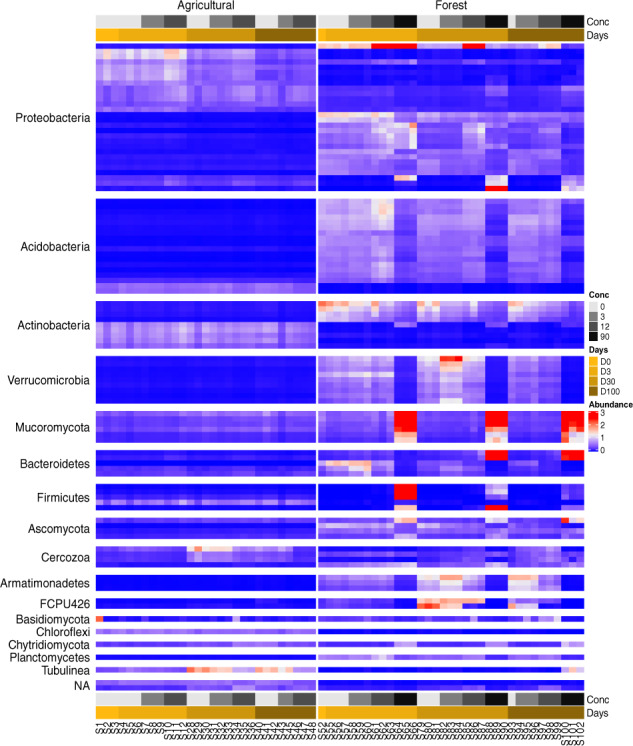
Heatmap of abundance distribution for top 100 most dominant contigs among samples (*n* = 100 K). Samples are ordered at increasing incubation times and wood ash amendment

MetaRib is able to recover more rRNA contigs across all domains and captures more taxa than before. For example, the fungal division *Mucoromycota* appears to be dominant in both with an abundance of approximately 3.5% in Forest at the highest ash concentration, while missing in the previous analysis (Bang-Andreasen *et al.*, 2020) (Fig. 7). MetaRib also allowed us to carry out taxonomy-independent statistics that were not possible when assembling reads sample-by-sample. Thus, we observed several interesting abundance patterns among the top 100 dominant contigs, illustrated as a heatmap in Figure 7. For example, while *Proteobacteria* were ubiquitous in both soils, different contigs dominated and showed more fluctuations in the forest. Contigs affiliated to the *Acidobacteria* were dominant in the forest soil and most of their abundances were positively correlated with concentration; however, they dropped significantly at the highest ash concentration. Besides, one *Firmicutes* affiliated contig was only presented in agricultural soil, while other *Firmicutes* contigs were only abundant in the highest dose in forest soil. *Verrucomicrobia* associated contigs showed the opposite trend.

## 4 Discussion

Here, we present the tool MetaRib for reconstructing rRNA genes from large scale total RNA meta-transcriptomic data. Its main advantage compared to existing methods is to quickly and reliably assemble rRNA contigs across multiple samples, even in very large datasets, with a low false positive rate and a taxonomy-independent relative abundance estimation.

Using simulated datasets, we show that MetaRib performs similarly to EMIRGE (representing the current state-of-the-art) in terms of recovering the underlying full-length true sequences, at the same time avoiding generating as many unreliable sequences (false positives) with a significant speedup. Besides, it provides an opportunity to have an overview of the abundance distribution across multiple samples, which could indicate important functions or patterns when combined with biological information.

Still, some challenges remain. Both EMIRGE and MetaRib are reference-based approaches, which could have issues in recovering novel and similar contigs when there is lacking information in the reference database (Datasets **b** and **c**): only partial sequences could be reconstructed in such extreme scenario. The contrasting results of simulated datasets indicate that MetaRib is able to capture most information in relatively well-characterized environments while it is more likely to generate false positives and partial sequences for poorly characterized environments. It also illustrates that the reference database is crucial for performance. While the most recent release of Silva includes over 9 million SSU sequences, our simulations used a less inclusive, earlier version, clustered at 94% sequence identity. It is likely that a more recent version will result in higher similarity for rRNA sequences, but it also result in longer execution times. At any rate, a non-redundant reference database is recommended, since EMIRGE is limited to reconstructing sequences with maximum 97% similarity to each other (Miller *et al.*, 2013). Other recent tools for rRNA assembly such as MATAM (Pericard *et al.*, 2018) have been shown to perform better than EMIRGE on small datasets, and future work could include using MATAM within the MetaRib tool.

An advantage of total RNA meta-transcriptomics is the ability to estimate relative abundances of rRNA sequences as proxies of microbial taxa, without PCR bias. Similarly, applications of third-generation sequencing like Oxford nanopore also have this advantage together with extreme long sequencing reads and real-time identification, which has shown great potential in microbial research (Jain *et al.*, 2016; Shin *et al.*, 2016).

However, it is important to point out that the number of rRNA reads does not represent an unbiased estimate of neither the metabolic activity nor the abundance (biomass or cell numbers) of the taxa as such, since rRNA gene copy number and patterns of ribosomal transcription and retention vary between organisms (Blazewicz *et al.*, 2013). In addition, so far it seems to be no commercial kit from Oxford Nanopore for sequencing of prokaryotic or total RNA, only eukaryotic, polyA-tagged mRNA sequencing.

Several parameter settings will also impact the performance of MetaRib, especially for large scale datasets, as illustrated here using a real-world dataset. In particular, the trade-off between execution time and the quality of the final results needs to be considered carefully. For example, increasing the read subsampling number will lead to longer execution times, but generate more low abundance contigs from rare organisms, thus recovering more of the diversity. However, it also leads to more false positives in terms of incorrectly assembled contigs.

In the current implementation, MetaRib discards any remaining unmapped reads after the iteration process is finished. However, taxonomy-independent rRNA assembly tools like REAGO could be considered as a further step to assemble discarded reads in order to maximize the information recovered from total RNA datasets.

Our approach opens up several new perspectives for total RNA meta-transcriptomics. First of all, it simplifies the analysis of the large and redundant datasets generated, via iterative reconstruction. In doing so, it also reduces false positives and allows for taxonomy-independent comparisons of contig abundances across samples. In spite of its advantages, total RNA has not been widely used compared to other environmental genomics techniques We hope that MetaRib will enable researchers to make more use of this technique and the valuable rRNA sequence data generated, with full-length sequences free of primer bias. Ultimately, this enables a deeper understanding of how natural microbial communities are structured, as well their function.

## Financial Support

none declared.

## Conflict of Interest

none declared.

## Supplementary Material

btaa177_Supplementary_Data
