# Supplementary material for: Reconstructing ribosomal genes from large scale total RNA meta-transcriptomic data
Source: Bioinformatics. 2020 Mar 13;36(11):3365–71. doi: 10.1093/bioinformatics/btaa177 (PMC7267836; doi:10.1093/bioinformatics/btaa177)
Supplement: btaa177_Supplementary_Data [file btaa177_supplementary_data.zip › btaa177-Suppl_Data/Supplementary Figures.docx]

**Supplementary Figures**

Fig. S1. Histogram of global pair wise similarity distribution of each dataset.

Fig. S2. Histogram of contig length distribution between source (SRC), MetaRib and EMIRGE using log10 transformation for both axes.
